# The evolving landscape of predictive biomarkers in immuno‐oncology with a focus on spatial technologies

**DOI:** 10.1002/cti2.1215

**Published:** 2020-11-22

**Authors:** Habib Sadeghi Rad, Sajad Razavi Bazaz, James Monkman, Majid Ebrahimi Warkiani, Nima Rezaei, Ken O'Byrne, Arutha Kulasinghe

**Affiliations:** ^1^ School of Medicine Tehran University of Medical Sciences Tehran Iran; ^2^ School of Biomedical Engineering University of Technology Sydney Sydney NSW Australia; ^3^ The School of Biomedical Science, Institute of Health and Biomedical Innovation Queensland University of Technology Brisbane QLD Australia; ^4^ Translational Research Institute Woolloongabba QLD Australia; ^5^ Institute of Molecular Medicine Sechenov University Moscow Russia; ^6^ Network of Immunity in Infection, Malignancy and Autoimmunity (NIIMA) Universal Scientific Education and Research Network (USERN) Tehran Iran; ^7^ Research Center for Immunodeficiencies Children's Medical Center Tehran University of Medical Sciences Tehran Iran; ^8^ Princess Alexandra Hospital Woolloongabba QLD Australia; ^9^ Institute for Molecular Biosciences University of Queensland Brisbane QLD Australia

**Keywords:** digital spatial profiling, immunotherapy, spatial profiling, tumor microenvironment

## Abstract

Immunotherapies have shown long‐lasting and unparalleled responses for cancer patients compared to conventional therapy. However, they seem to only be effective in a subset of patients. Therefore, it has become evident that a greater understanding of the tumor microenvironment (TME) is required to understand the nuances which may be at play for a favorable outcome to therapy. The immune contexture of the TME is an important factor in dictating how well a tumor may respond to immune checkpoint inhibitors. While traditional immunohistochemistry techniques allow for the profiling of cells in the tumor, this is often lost when tumors are analysed using bulk tissue genomic approaches. Moreover, the actual cellular proportions, cellular heterogeneity and deeper spatial distribution are lacking in characterisation. Advances in tissue interrogation technologies have given rise to spatially resolved characterisation of the TME. This review aims to provide an overview of the current methodologies that are used to profile the TME, which may provide insights into the immunopathology associated with a favorable outcome to immunotherapy.

## Graphical abstract

In this review, we discuss spatial analysis tools that can be used to characterise the tumor microenvironment. We present a number of technologies that are transforming the field and providing insights into the immunopathology associated with a favorable outcome to immunotherapy.

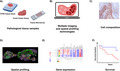

## Introduction

Immune checkpoint inhibitors (ICIs) are a form of immunotherapy and have offered long‐lasting and durable benefits to a number of tumor types including melanoma, bladder, head and neck and lung cancer.^1^ However, ICI therapy only appears to benefit a subset of patients. Therefore, there is an unmet clinical need to identify biomarkers predictive of outcome to therapy. It is thought that a greater understanding of the immune contexture (cell type, density, function) in the tumor microenvironment (TME) may shed light upon tissue activation and immune recognition, which in turn may be used to predict response to therapy.^2^ The TME encloses the cell types and vascularisation in forming the tumor, including blood and lymphoid vessels, the extracellular matrix, and immune infiltrates.^1^ As such, tumor growth, invasion and resistance to therapy derive from bidirectional interaction between the tumors and the microenvironment.^3^ Therefore, having knowledge of the co‐evolution of tumors and their immediate microenvironment in order to understand the underlying tumor‐immune cell interactions, the degree of tumor cell recognition, and the types of cells recruited into the microenvironment is needed to develop more effective therapies.^4^ The TME is composed of a complex milieu of cell types.^5^ Bulk analysis of the tumor either at the protein/transcript level is not sufficient to capture a spatially resolved representation of the TME.^5^ To address this, advances in multiplex immunohistochemistry, imaging and barcoding methodologies have led to tools which enable the phenotyping of the TME for composition, function, activity and spatial location of cells.^6^ In this review, we will provide an overview of the current TME biomarkers used as predictive biomarkers of therapy and provide insights into technologies which are able to spatially map the TME.

## Predictive biomarkers of response to immunotherapy

In recent years, immunotherapies, in particular ICIs, have been employed to reinvigorate anti‐tumor response and have changed clinical cancer care.^7^ This was led by the humanised anti‐cytotoxic T lymphocyte antigen 4 (CTLA‐4) antibody ipilimumab in 2011, which has been found to double 10‐year survival for patients with metastatic melanoma compared to conventional therapies.^8^, ^9^ Thereafter, blockade of another checkpoint protein, programmed cell death 1 (PD1), or its ligand, PD1 ligand 1 (PD‐L1), has been shown to have even higher response rates and lower incidence of side effects relative to anti‐CTLA‐4.^10^, ^11^ Tumor cells upregulate PD‐L1 in order to evade immune responses.^12^ PD‐L1 expressed on tumor cells interacts with its receptor PD‐1 on T cells to prevent T‐cell activation.^13^ Therefore, the expression of PD‐L1 is a mechanism by which tumor cells can inhibit T‐cell immunity.^14^ The United States Food and Drug Administration (US FDA) has approved antibodies targeting the PD1–PD‐L1 axis as first‐line or second‐line therapies for a number of cancers, including melanoma, lung cancer, lymphoma, head and neck squamous cell carcinoma (HNSCC), renal cell cancer (RCC), gastro‐oesophageal cancer, and liver cancer.^15^ While promising, it appears that only some patients benefit from this therapy. Therefore, there is a need to develop predictive biomarkers of ICI therapy, in order to personalise therapy to the individual patient.

## PD‐L1 expression

Programmed cell death 1‐ligand 1 is assessed using immunohistochemistry (IHC) and scored by a pathologist.^16^, ^17^ The US FDA has approved the IHC assay for PD‐L1 protein expression as a companion/complementary diagnostic marker for anti‐PD‐L1 therapy.^16^, ^17^ As such, tumor PD‐L1 expression has been known to be the most widely used predictive biomarker of ICI response.^18^ However, the use of PD‐L1 expression as a predictive biomarker has been challenging because PD‐L1 has a dynamic level of expression.^19^, ^20^ The dynamic nature of PD‐L1 is further compounded by poor uniformity in PD‐L1 IHC antibodies, variable cut‐offs and tumor/immune cell type levels of expression which corresponds to benefit to therapy.^14^, ^21^ Moreover, it has been shown that the levels of PD‐L1 expression are transient and change over time; therefore, timing of the biopsy may be crucial too.^22^ A number of studies have shown that the expression of PD‐L1 on tumor cells could be used as a predictive marker,^23^, ^24^ while others have suggested that PD‐L1 expression on tumor‐infiltrating immune cells, such as macrophages, is more informative.^18^, ^25^ A study by Rimm *et al*. in triple‐negative breast cancer (TNBC) treated with a combination of immunotherapy (durvalumab) and chemotherapy (the Impassion 130 and the Keynote 522 trials) examined PD‐L1 expression on both tumor and immune cells.^26^ The findings showed that patients with the pathologic complete response (pCR) were shown to have higher PD‐L1 expression in tumor, stromal and CD68 (macrophage) compartments compared to patients with non‐pCR.^26^ Therefore, a more comprehensive characterisation of the immune contexture is needed to gain insights into the surrounding structures and cell types that are recruited into that area. This may give insights into which patients respond to immunotherapy more readily than a single marker expression such as PD‐L1.^14^ Therefore, the expression of PD‐L1 on both tumor cells and macrophages was associated with the immunotherapy and chemotherapy combination response.^26^

## Tumor mutational burden

Genomic aberrations including point mutation and small insertion/deletion (indel) have been found to generate neoantigens, which result in the induction of the host immune response.^27^ The assessment of the mutational landscape of the tumor has been commonly termed ‘tumor mutational burden’ (TMB).^28^ TMB is defined as the number of somatic mutations per DNA megabase (Mb) and is used as a genomic biomarker for the identification of patients likely to respond to immunotherapy.^28^ Studies have shown that patients with high TMB are more likely to benefit from immunotherapy agents due to the increased rate of immunogenicity.^29^, ^30^ The KEYNOTE‐158 study recently reported that patients with TMB‐High (TMB‐H), ≥ 10 mut Mb^−1^ (mutation/megabase), had an improved overall survival following treatment with pembrolizumab (anti‐PD1 antibody, KEYTRUDA), than those with a TMB less than 10 mut Mb^−1^.^30^ This result was found in patients across multiple tumor types, including anal, biliary, cervical, endometrial, salivary, thyroid, vulvar, mesothelioma, neuroendocrine and small‐cell lung cancer (SCLC). The study showed that TMB‐H patients with unresectable or metastatic solid tumors could benefit from KEYTRUDA, in a tissue agnostic manner.^30^

In addition to taking into account the quantity of TMB, the quality of the mutations should also be considered. This means that certain forms of mutations are more likely to induce an immune response. For instance, it has been found that indel mutations could lead to higher immunogenicity in comparison with missense mutations.^31^ Clinical responses in patients with a defect in the DNA mismatch repair mechanism (MMR) have been reported to be associated with the indel mutational load but not with the missense mutation.^31^ Furthermore, studies have shown that mutations in specific genes may result in outcomes to immunotherapy.^32^ In patients with mutations in the interferon‐gamma receptor (IFNGR) signalling pathway, such as tyrosine‐protein kinase JAK1 (JAK1), JAK2, and apelin receptor (APLNR), have been shown to be resistant to therapy.^33^ Moreover, it has been demonstrated that specific human leukocyte antigen (HLA‐I) serotypes can have a significant role in response to therapy.^34^ It has been found that patients with HLA B44 and B62 serotypes could benefit from ICI antibodies.^34^

Tumor mutational burden was originally measured using whole‐exome sequencing (WES), and a number of studies reported an association between WES‐derived TMB and response to ICIs.^28^, ^35^ WES‐derived TMB measurement requires the matched normal sample can be time‐consuming to perform.^36^ Therefore, to overcome this, next‐generation sequencing (NGS)‐based panels that sequence a sufficient subset of the exome have been developed for calculating TMB.^37^, ^38^, ^39^ In addition to time and cost‐effective advantages, the targeted NGS panels take into account both nonsynonymous and synonymous base substitutions as well as short insertion/deletion alterations.^38^ These inclusions for the TMB measurement have led to an improved assay sensitivity by increasing the number of qualifying variants into the calculation.^38^ For this, there are currently two FDA‐approved NGS panels for calculating TMB: the FoundationOne CDx and MSK‐IMPACT (Memorial Sloan Kettering‐Integrated Mutation Profiling of Actionable Cancer Targets) panels.^7^ These panels have been designed to detect a number of DNA alterations, including point mutations, small and large insertions/deletions, copy number variations, and structural variants, in cancer‐related genes.^36^ These assays have given insights into microsatellite instability (MSI), loss of heterozygosity, and TMB. Further studies are warranted to investigate the role of TMB as a predictive biomarker and harmonisation between NGS‐based TMB assays.^39^ The project known as the *Friends* TMB harmonisation project was designed to establish a uniform approach for the measurement and reporting of TMB across various sequencing panels. The project consists of three phases, *in silico*, empirical and clinical analyses.^40^ These analyses use publicly available TCGA data, cells derived from human tumors, and human FFPE tumor samples, to harmonise the definition of TMB and to ensure consistency in the calculation of TMB through alignment with a universal reference standard.^40^ The first two phases have been completed. The *in silico* analysis has indicated that the higher WES‐TMB value, the greater the variability within and between panel TMB values. Certain types of cancers, including uterine, bladder and colon cancers, have shown greater variability in panel TMB values relative to others, such as lung and head and neck cancers.^40^ The empirical analysis has also shown that the variability across laboratories tends to increase in line with the increase in the WES‐TMB values. Finally, future studies (e.g. clinical analyses) will focus on the use of samples from ICI‐treated patients to evaluate optimal cut‐off values that help guide the clinical application of TMB.

## Microsatellite instability

Microsatellites (MSs) are short tandem DNA sequences (usually 1–6 nucleotides long) repeated throughout the genome.^41^ These sequences are located in both genes and inter‐gene regions, often present in promoter, exons, introns and untranslated terminal regions (UTRs).^42^ If the repetition of these sequences changes, increases or decreases, there will be MS instability. Such errors are usually corrected by the DNA repair mechanism known as the mismatch repair (MMR) system.^43^ The MMR system consists of key proteins such as MLH1, MSH2, PMS2 and MSH6; therefore, mutations in any of these genes, either germline or somatic, could cause a defect in the MMR mechanism and termed ‘MMR deficiency’.^44^ MMR deficiency contributes to the generation of many indel mutations.^45^ A number of indel mutations may lead to frameshifts in the DNA sequences that produce neoantigens with more immunogenic characteristics.^7^ It has been shown that MSI‐positive tumors, such as colorectal cancers (CRCs), are highly CD8^+^ T cell infiltrated compared with microsatellite stable counterparts.^46^ This finding could explain as to why MSI‐positive tumors show high objective response rates to ICIs.^7^ Pembrolizumab has been approved by the FDA for the treatment of MSI‐high/MMR‐deficient tumors. There are currently two approaches to detecting MSI‐high and MMR‐deficient tumors in clinics, polymerase chain reaction (PCR) and IHC.^47^ Although MSI‐high has been reported in multiple solid tumors, it varies across different types of tumors.^48^ CRC, endometrial and gastric cancers have the highest frequencies (> 10%), while glioblastomas, oesophageal cancer, breast cancer and non‐small‐cell lung cancer (NSCLC) have the lowest frequencies (< 2%).^48^ Thus, despite the fact that MSI can enhance neoantigen load and induce a better response to ICIs, its low frequencies in human tumors restrict its application to immunotherapy as a broad‐based predictive biomarker.^49^

## Tumor microenvironment

The TME is known to play a key role in the initiation and progression of cancer.^4^, ^50^ There are a variety of host immune cells recruited within the TME. These include cells that are involved in both the innate and adaptive immune responses.^51^ which may act as a tumor promoter, and some may suppress the tumor.^52^ Thus, the immune contexture, that is the type, density and location of cells in the TME, could be useful to understand the underlying biology associated with a favorable treatment outcomes.^4^ Immune infiltration of tumors is classified as immune‐inflamed, immune‐excluded, and immune‐desert.^7^ The immune‐inflamed is when there are CD3^+^ and CD8^+^ T cells in the tumor regions and the invasive margin, while an immune‐desert represents a low density of both cell types in both regions.^53^ The immune‐excluded phenotype reflects the presence of T cells at the invasive margin without the ability to infiltrate the tumor.^53^ It has been shown that the inflamed TME is usually accompanied by the expression of immune checkpoint proteins such as PD‐L1 on infiltrating immune cells (e.g. macrophages) and tumor cells, suggesting that these types of tumors have pre‐existing anti‐tumor immune responses.^54^ Tumors with this type of TME are therefore more likely to respond to PD‐1/PD‐L1 blockade.^55^ This function allows for inflammatory gene signatures such as IFN‐ γ signalling genes to be used as ICI biomarkers to select appropriate patients for therapy.^56^ Moreover, studies have reported that the presence of transforming growth factor‐β (TGF‐β) signalling pathway can contribute to the exclusion of CD8^+^ T cells from the tumor parenchyma.^57^ Blocking the TGF‐β signalling pathway could have the potential to convert the TME to a more inflamed state and make it more susceptible to ICIs.^57^

## Tertiary lymphoid structures

For an anti‐cancer immune response to generate efficiently, dendritic cells (DCs) are required to migrate from the tumor site to secondary lymphoid organs (SLOs) and present major histocompatibility complex (MHC) molecule–peptide antigen complexes to CD4^+^ and CD8^+^ T cells.^58^, ^59^, ^60^, ^61^ Within the follicles of SLOs, B cells are also activated and undergo proliferation, isotype switching, and somatic hypermutation.^62^ Recognition of antigens presented by DCs and receiving co‐stimulating signals provided by the CD4^+^ T cells are required for this.^62^ This results in the proliferation and differentiation of lymphocytes to effector T cells and memory B cells, which eventually infiltrate the tumor mass and kill the tumor cells.^63^ The generation and regulation of immune response to tumor cells not only occur in SLOs but can also occur directly at the tumor site, in tertiary lymphoid structures (TLSs).^63^ TLS represents lymphoid neogenesis caused by long‐lasting exposure to inflammatory signals mediated by chemokines and cytokines.^64^ TLS develops under the influence of various pathophysiological conditions, such as autoimmune diseases and cancer, and their function is context‐dependent.^64^ This structure is also composed of a variety of immune cells, including B and plasma cells, CD4^+^ and CD8^+^ T cells, DCs, macrophages and neutrophils.^65^ TLS provides the conditions for DCs to present adjacent tumor antigens to T cells, activation, proliferation and differentiation of B and T cells.^65^As the density of TLS is associated with the presence of CD4^+^ and CD8^+^ T cells in tumors, its development is related to a favorable prognosis in cancer patients.^66^ TLS can develop in the stroma, invasive margins and the core of the tumor mass; however, its abundance in stroma or invasive margins is higher than the core of tumors.^67^

## Spatial tumor microenvironment profiling technologies

Spatial and immunological composition with cellular status can aid in identifying micro‐niches within the TME. The classification of the immune context within the TME lays the foundation to addressing how the immunological composition and status (activated/suppressed) may dictate response to therapy. Therefore, to address this need, imaging and tissue sampling is required simultaneously to analyse tumor tissue and immune proteins with spatial resolution (Table 1). Studies have demonstrated using multiplex immunofluorescence that the proximity of immune and tumor cells in the TME underlies the response to anti‐PD‐1 targeted therapies.^68^

**Table 1:** Overview and comparison of spatial transcriptomics profiling technologies

Technology	Sample type	Resolution	Approach	Analyte	Advantages/Disadvantages
Imaging Mass Spec (IMC)	Fresh‐frozen FFPE	Cellular Subcellular	Metal‐based	Peptides Protein	*Pros*: Molecular analysis retaining spatial distribution of analytes, 2D distribution maps for each mass measured *Cons*: Sample preparation, low throughput, data processing and analysis
10x Chromium	Fresh‐frozen	Cellular	Barcoded Gel Beads	RNA	*Pros*: Whole transcriptome *Cons*: No spatial resolution
10x Visium	Fresh‐frozen	Anatomical features of 100 µm/55 µm Cellular	Barcoded mRNA capture spots	RNA	*Pros*: Whole transcriptome *Cons*: Barcoded regions contain multiple cells
CODEX	Fresh‐frozen FFPE	Cellular	DNA‐barcoding‐based	Protein	*Pros*: Allows the analysis up to 40 proteins, spatial and single‐cell resolution. *Cons*: Whole slide can be time‐consuming/costly
NanoString GeoMX DSP	Fresh‐frozen FFPE	Custom down to 10 µm Cellular	DNA‐barcoding‐based	RNA Protein	*Pros*: 96–20000 mRNA detection (whole transcriptome) High level of automation *Cons*: No image reconstruction Requires manual choice of regions
Ultivue	Fresh‐frozen FFPE	Cellular Subcellular	DNA‐barcoding‐based	Protein	*Pros*: Whole‐slide multiplexing and imaging Rapid and automated workflow *Cons*: No slide scanner

## Imaging mass cytometry

Imaging mass cytometry (IMC) is a type of mass cytometry combined with a novel laser ablation system that quantifies the expression of multiple markers with subcellular spatial resolution on a single tissue section.^69^ IMC not only provides in situ spatial information and antigen qualification but can also be performed in both snap‐frozen and formalin‐fixed paraffin‐embedded (FFPE) tissue sections.^70^ Therefore, IMC contributes to the simultaneous characterisation of the composition of the immune compartment, revealing the spatial relationship between immune cells and stromal cells, and demonstrating interactions among immune subsets in tissue areas of preference.^69^ Unlike classical immunohistochemistry or immunofluorescence techniques, which suffer from background interference due to the use of a limited number of markers, IMC takes advantage of rare metals conjugated to antibodies to significantly improve the multiplexing capacity.^71^ Tissue sections are labelled with multiple antibodies conjugated to stable isotopes and then ablated with a laser system to create segments of 1 μm in diameter.^71^ When inserted into the mass cytometer, atomised and ionised, the metal‐isotope content of each segment is measured by the time‐of‐flight mass analyzer.^70^ Finally, the isotope abundance of each spot is used to produce a high‐dimensional image.^70^ In the study by Ali *et al*., IMC was used to quantify protein expression with subcellular resolution in multiple tumor tissues to investigate the impact of somatic alterations on the tumor ecosystems. Phenotypes and cell–cell interactions were shown to be associated with genomic subtypes of breast cancer, with those expressing Ki67 and HER2 associated with poorer outcomes.^72^ Aoki *et al*. used IMC to characterise immune cell populations to generate an immune cell atlas for the TME of Hodgkin lymphoma (HL). A novel subtype of T cells with expression of the inhibitory receptor lymphocyte activation gene 3 protein (LAG3) was identified which acted as a mediator of immunosuppression. In the study, increased LAG3^+^ T cells were shown to be in the direct vicinity of MHC class‐II‐deficient tumor cells.^73^ Zhu *et al*. investigated ovarian cancer patients using IMC to identify biomarkers of immunotherapy response. In the study, the authors found that the highest increase in CD8^+^ T cells and forkhead box protein P3 (FoxP3)^+^ cells was found in patients responding to the combination of durvalumab and tremelimumab.^74^

## 10x Genomics

### Chromium single‐cell gene expression

Analysis of cell‐type variations in biological systems is crucial to the understanding of the cellular contribution to cancer progression.^61^ The Chromium Single‐cell Gene Expression Solution with Feature Barcoding Technology (with Next GEM technology) utilises both cell surface protein detection and single‐cell transcriptome readout.^75^ This technology takes advantage of antibodies conjugated to DNA barcodes for single‐cell sequencing.^76^ Using Feature Barcoding technology, it would be plausible to analyse multiple markers in a single assay.^76^ These characteristics improve the resolution of the cell type, therefore, leading to the detection of rare cell types, and the discovery of more unique transcripts all in a single assay at single‐cell resolution.^75^ Andor *et al*.^77^ used the 10x Chromium to obtain single‐cell transcriptomes of follicular lymphoma (FL). The study found that malignant B cells exhibited a downregulation of the FCER2, CD52 and MHC class II genes. T cells in the FL tumors expressed high levels of immune checkpoint genes.^77^ Additionally, Zhang *et al*.^78^ used 10x Chromium to characterise a single‐cell profile of early gastric cancer (EGC). A panel of EGC‐specific signature with clinical implications for the diagnosis of EGC was identified.^78^ This panel consisted of the genes in kallikrein‐10 (KLK10), natural resistance‐associated macrophage protein 2 (SLC11A2), sulfotransferase 2B1 (SULT2B1), kallikrein‐7 (KLK7), extracellular matrix protein 1 (ECM1) and LMTK3.^78^

### Visium spatial gene expression

The Visium Spatial Gene Expression solution is used to measure transcripts and gene expression across a tissue section.^79^ The Visium technology has the ability to be combined with immunofluorescence in order to visualise protein and gene expression at the same time.^5^ The technology is compatible with fresh‐frozen samples from most tissue types and uses thousands of barcoded mRNA capture spots to visualise gene expression with both whole transcriptome analysis and targeted gene expression panels.^79^ Visium provides insights into the relationship between cell function, phenotype and location in tissue microenvironments by preserving the spatial context of tissues along with the identification of distinct groups of cells.^5^ Such technology could, therefore, provide clinical applications, including insight into tumor heterogeneity and tissue morphology, identification of response to therapeutic interventions, and discovery of biomarkers.^79^ In the study by Ji Al *et al*., Visium was used to define the cellular composition and architecture of cutaneous squamous cell carcinoma (cSCC). It was shown that among multiple cell types in the cSCC, tumor‐specific keratinocytes (TSKs) acted as a hub for intercellular communication. TSKs were also found to reside within a fibrovascular niche at leading edges.^80^ Moreover, the study found that TSK, basal and adjacent stromal and immune cell types exhibited invasive and immunosuppressive characteristics associated with physical proximity and distinct sets of ligands and receptors.^80^

## CO‐Detection by antibody indEXing (CODEX, Akoya Biosciences)

CO‐Detection by antibody indEXing (CODEX, Akoya Biosciences, Menlo Park, CA, USA) is a type of multiplex fluorescence microscopy platform using DNA‐conjugated antibodies that allows analysis of up to 40 targets in a single tissue section. The CODEX platform can recognise single cells in their tissue, as well as discover novel cell types and cell–cell interactions.^81^ Unlike other cyclic immunofluorescence (CycIF) approaches which have several antibody staining and stripping steps, the CODEX platform employs a single initial staining step and subsequent manipulation of tissues, resulting in a rapid workflow and preventing tissue degradation. Using complementary fluorescent DNA probes, DNA‐conjugated antibodies are made visible, accompanied by imaging, probe stripping, washing and re‐rendering. Phillips *et al*. used the CODEX platform to evaluate the response to immunotherapy in cutaneous T‐cell lymphoma (CTCL). In patients who responded to pembrolizumab, the effector‐type cellular neighbourhoods (CNs), including a tumor/DC CN and a tumor/CD4^+^ T‐cell CN, were significantly increased, while in non‐responder an immunosuppressive‐type CN enriched in regulatory T cells was significantly increased following treatment. Schürch *et al*.^82^ identified spatially nuanced interactions between components of the immune TME. Nine conserved, distinct CNs were identified in colorectal cancer (CRC) TME. Enrichment of PD‐1^+^CD4^+^ T cells only within a granulocyte CN had a positive correlation with survival in a high‐risk patient subset.^84^ Worse outcomes were associated with the combination of tumor and immune CNs, fragmentation of T cell and macrophage CNs, and disruption of inter‐CN communication.^82^

## NanoString GeoMx™ digital spatial profiler

**Figure 1:**
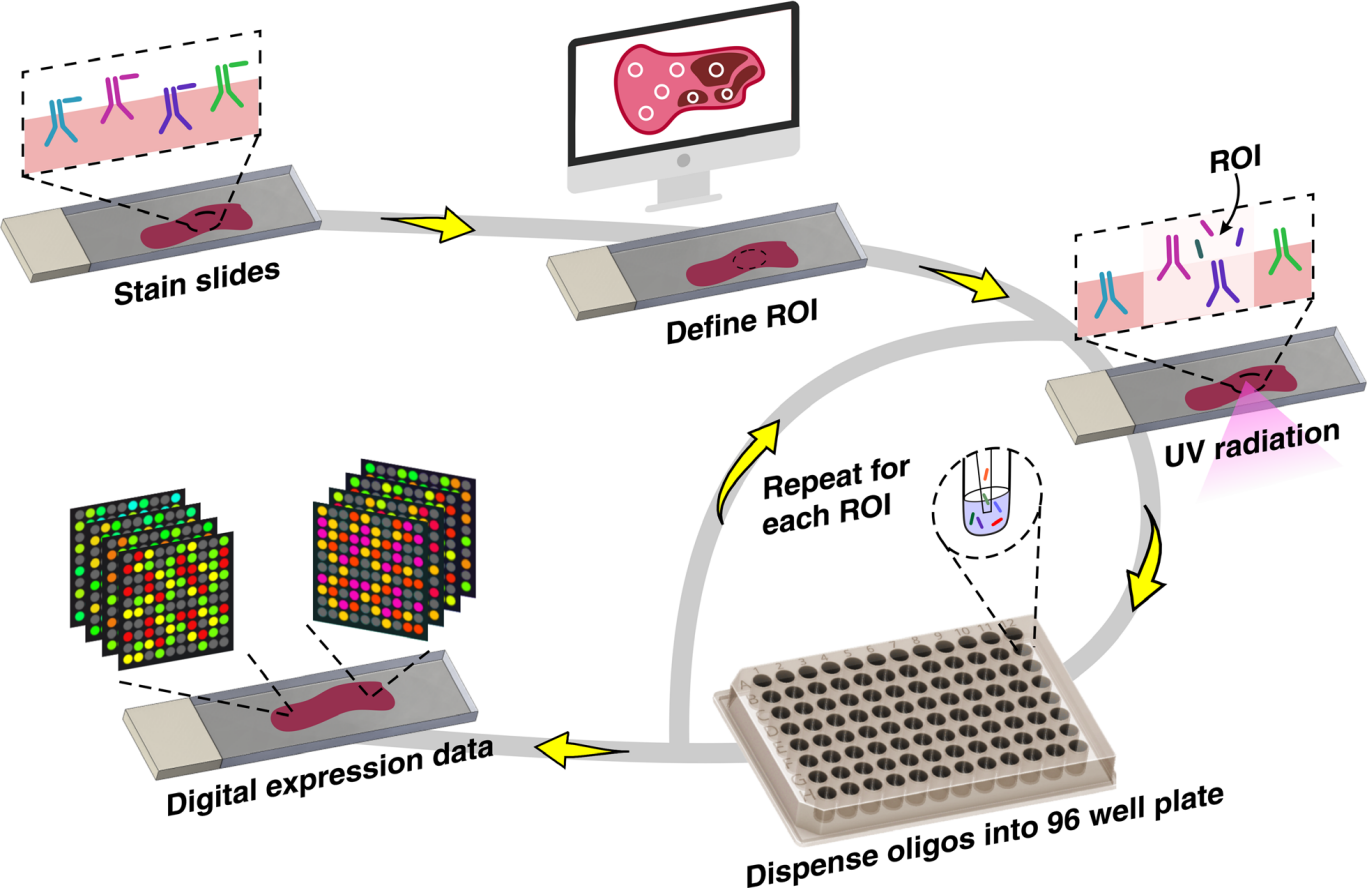
NanoString GeoMX Digital Spatial Profiler (DSP) workflow for interrogating multiple protein/RNA analytes from a single tissue section. Analytes in the tissue sections are conjugated with oligo tags via photocleavable linkers. The user defines regions of interest (ROI) from an initial visualisation to demarcate the tissue architecture. Then, spatially mapped UV illumination allows oligo tags to be released from the analyte into a 96‐well plate. The collected oligos are then subject to standard NanoString counting/sequencing to obtain digital counts per ROI.

The NanoString GeoMx™ Digital Spatial Profiler (DSP) with digital colour‐coded ‘barcodes’ is capable of detecting and quantifying protein and mRNA at significantly higher multiplex manner (40–100 protein and to 96–18 000 mRNA targets) from fixed and fresh‐frozen tissues with spatial resolution (Figure 1).^83^ Compared to other multi‐colour IHC techniques, the DSP retains tissue structure without degrading samples because as the UV‐photocleavable signal is liberated and counted, without the need for chemical stripping.^83^ The DSP has recently been used in multiple tumor types, such as melanoma, non‐small‐cell lung cancer (NSCLC), and renal cell carcinoma (RCC).^84^ Rimm *et al*. utilised the DSP technology to identify biomarkers associated with outcome to therapy in melanoma. They found that CD8, CD3, TIM3, HLADR, IDO1 and CD11c in tumor regions were associated with a favorable progression‐free survival (PFS). In patients with CD8, B2M, PD‐L1 and TIM3 present in macrophages and B2M in lymphocytes, had a better PFS.^18^ Monkman *et al*. characterised the TME of non‐small‐cell lung cancer (NSCLC). The study compared the TME and normal adjacent tissue (NAT) revealing that several proteins including CD34, fibronectin, IDO1, LAG3, arginase‐1 (ARG1) and PTEN were downregulated in TME relative to NAT. When the TME and tumor were compared, the study showed that CD3, CD45RO, V‐domain Ig suppressor of T‐cell activation (VISTA), and CD163 were enriched in TME relative to tumor.^84^ Wargo *et al*. showed a nine‐gene signature associated with tertiary lymphoid structures (TLSs) in melanoma. The gene signature included CD79B, CD1D, CCR6, linker for activation of T‐cell family member 1 (LAT), Src kinase‐associated phosphoprotein 1 (SKAP1), cholesteryl ester transfer protein (CETP), eukaryotic translation initiation factor 1A, Y‐chromosomal (EIF1AY), retinol‐binding protein 5 (RBP5) and prostaglandin‐H2 D‐isomerase (PTGDS).^60^ It was also shown that T cells had a dysfunctional molecular phenotype in tumors without TLS structures.^60^ In another study, Wargo *et al*., in tissue samples from patients with melanoma and renal cell carcinoma (RCC), showed that the density of CD20^+^ B cells and TLSs, as well as the ratio of TLS to tumor area, was higher in responders to immunotherapy than in non‐responders.^59^ In the responders, CD20^+^ B cells were located in TLSs of tumors and were co‐localised with CD4^+^, CD8^+^ and FoxP3^+^ T cells.^59^

## Ultivue

Multiplex IF assists visualisation of multiple biomarkers simultaneously in tissue while preserving the spatial context.^85^ Ultivue platform uses InSituPlex DNA‐barcoding and antibody staining technology to provide whole‐slide multiplexing for cell phenotyping in addition to the spatial profiling of tissue biomarkers. InSituPlex technology is capable of detecting multiple markers on single cells, even in the same cellular compartment.^85^ Markers can also be easily detected in a wide range of cellular compartments such as plasma membrane, cytoplasm and nucleus. This contributes to accurate and in‐depth immunophenotyping in tissues through positive detection of markers. InSituPlex technology enhances the number of hybridisation sites for imaging by using linear barcode amplification while controlling different levels of expression from marker‐to‐marker and cell‐to‐cell. This technology uses a gentle staining method to prevent the loss of integrity of the tissue sample. The platform provides high‐performance tissue multiplexing as well as multiple biomarker co‐localisation and co‐expression. Rimm *et al*., in NSCLC, showed that across tumor cells and multiple immune cells, the majority of PD‐L1 expression co‐localised with CD68^+^ cells.^25^ The expression of PD‐L1 in the macrophage compartment, but not in the tumor cell compartment, was also found to be associated with overall survival.^25^ Bleck *et al*.^86^ used Ultivue technology to capture complex immune cell phenotypes in FFPE samples from colorectal cancer (CRC) patients. The study found that hot CRC tumors were found to have an increase in PD‐L1^+^ CD68 cells relative to cold tumors. CD8^+^ T cells in the cold tumors were also found to be further away from the nearest PD‐L1^+^ cells compared to hot tumors.^86^ Moreover, Hutchinson *et al*. employed Ultivue platform to evaluate spatial immune infiltration patterns in CRC FFPE samples.^87^ As a result, it was demonstrated that high‐TMB tumors had a higher mean area of intraepithelial (IE) PD‐L1 and CD8, while low‐TMB counterparts had a higher mean area of IE CD68.^87^

## Concluding remarks and future perspectives

The TME is derived from a complex set of various cell types interacting with each other; however, it is unclear how these interactions cause the tumor cells to develop, proliferate and lead to metastasis. Multiple factors, including tumor type, tumor mutation burden, microsatellite instability, tertiary lymphoid structures and immune cell infiltration, have been shown to play a significant role in the TME and in turn inform on the response to immunotherapy. Consideration of the relationship between both these patients' intrinsic and tumor‐dependent effects at various levels will be critical to improving the efficacy of existing immunotherapeutic approaches. As such, a comprehensive and accurate understanding of the factors involved in the heterogeneity of the TME as well as the biological crosstalk of the tumor–host interface is crucial in promoting treatment strategies. To this end, multiplexed sequencing and imaging platforms that provide in situ and spatial information on various immune and non‐immune factors within the TME can significantly advance this field. Characterisation of the TME is a valuable method used for tumor subclassification and predicting clinical outcomes. Simultaneous quantification of multiple biomarkers using multiplexed spatial TME profiling technologies has become increasingly important. Spatial profiling technologies can provide comprehensive tissue, morphological, protein/gene expression analysis and insights into the tumor biology than has not been previously possible. Enabling deeper insights into the tumor–immune cell interactions and cellular interactions at play which may inform on outcome to immunotherapy.

## Conflict of Interest

The authors declare no conflict of interest.

## Author Contributions

**Habib Sadeghi Rad:** Writing‐original draft; Writing‐review & editing. **Sajad Razavi Bazaz:** Visualization; Writing‐original draft. **James Monkman:** Writing‐original draft; Writing‐review & editing. **Majid Warkiani:** Conceptualization; Visualization; Writing‐review & editing. **Nima Rezaei:** Writing‐review & editing. **Ken O'Byrne:** Conceptualization; Resources; Writing‐original draft; Writing‐review & editing. **Arutha Kulasinghe:** Conceptualization; Resources; Supervision; Writing‐original draft; Writing‐review & editing.
